# Folding for the Immune Synapse: CCT Chaperonin and the Cytoskeleton

**DOI:** 10.3389/fcell.2021.658460

**Published:** 2021-04-12

**Authors:** Noa Beatriz Martín-Cófreces, José María Valpuesta, Francisco Sánchez-Madrid

**Affiliations:** ^1^Immunology Service, Hospital Universitario de la Princesa, Universidad Autonoma Madrid (UAM), Instituto Investigacion Sanitaria-Instituto Princesa (IIS-IP), Madrid, Spain; ^2^Area of Vascular Pathophysiology, Laboratory of Intercellular Communication, Fundación Centro Nacional de Investigaciones Cardiovasculares-Carlos III, Madrid, Spain; ^3^Centro de Investigación Biomédica en Red de Enfermedades Cardiovasculares (CIBERCV), Madrid, Spain; ^4^Centro Nacional de Biotecnología (CNB-CSIC), Madrid, Spain

**Keywords:** CCT, chaperonin, immune synapse, tubulin, actin, cryocorrelative microscopy, microtubule

## Abstract

Lymphocytes rearrange their shape, membrane receptors and organelles during cognate contacts with antigen-presenting cells (APCs). Activation of T cells by APCs through pMHC-TCR/CD3 interaction (peptide-major histocompatibility complex-T cell receptor/CD3 complexes) involves different steps that lead to the reorganization of the cytoskeleton and organelles and, eventually, activation of nuclear factors allowing transcription and ultimately, replication and cell division. Both the positioning of the lymphocyte centrosome in close proximity to the APC and the nucleation of a dense microtubule network beneath the plasma membrane from the centrosome support the T cell’s intracellular polarity. Signaling from the TCR is facilitated by this traffic, which constitutes an important pathway for regulation of T cell activation. The coordinated enrichment upon T cell stimulation of the chaperonin CCT (chaperonin-containing tailless complex polypeptide 1; also termed TRiC) and tubulins at the centrosome area support polarized tubulin polymerization and T cell activation. The proteasome is also enriched in the centrosome of activated T cells, providing a mechanism to balance local protein synthesis and degradation. CCT assists the folding of proteins coming from *de novo* synthesis, therefore favoring mRNA translation. The functional role of this chaperonin in regulating cytoskeletal composition and dynamics at the immune synapse is discussed.

## Introduction

Synaptic contacts involve cell-cell communication structures determined by the polarization of organelles and specific cell components allowing the interchange of information, such as neurotransmitters, cytokines and genetic information, based on cytoskeleton rails (Martin-Cofreces et al., 2014). The immune synapse (IS) is a transient, dynamic cell-to-cell communication structure that forms at the interface of T cells and antigen-presenting cells (APCs). It represents a signaling hub, facilitating the sensing of extracellular cues to enable both T cell activation and differentiation and APC reprogramming/reorganization (Martin-Cofreces et al., 2014; Mastrogiovanni et al., 2020). On the T cell side, the changes to the cytoskeleton in response to T cell receptor (TCR) activation have been studied in the context of intracellular reorganization and, more recently, propagation of intracellular signals. An unresolved question is how the actin and tubulin cytoskeletons coordinate to rearrange both spatially and temporally. These two cytoskeletons are inter-connected through proteins that are able to physically link them, as well as by signaling proteins that control their dynamics, such as members of the protein kinase C (PKC) family, phospholipase C and formins such as INF2 (Quann et al., 2011; Andres-Delgado et al., 2012; Kumari et al., 2015; Murugesan et al., 2016). The interdependence of tubulin and actin dynamics and the occupancy of specific regions in the cell have been described in diverse contexts, mainly in highly differentiated cells such as neurons and immune cells that form synapses (Martin-Cofreces et al., 2014; Coles and Bradke, 2015).

**FIGURE 1:**
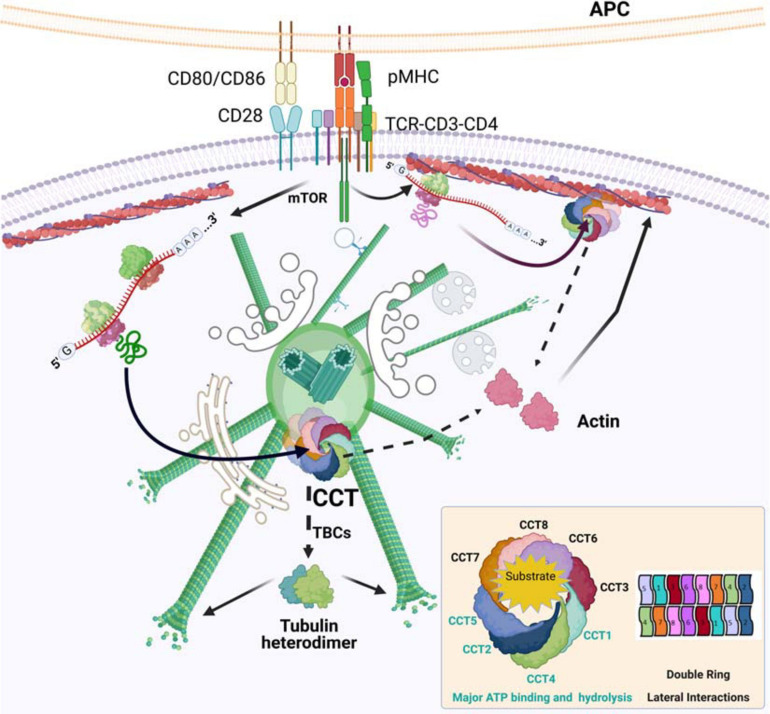
CCT in the reorganization of actin and tubulin at the immune synapse. TCR activation promotes protein synthesis. The chaperone CCT accumulates in activated centrosomes, which then can act as folding centers for newly synthesized proteins. The new polypeptides are assisted in their folding by CCT, whose major obligate substrates are actin and tubulin. Actin is directly sorted into its native form, whereas tubulin needs the assistance of different tubulin-binding co-factors (TBCs) to form stable αβ-heterodimers. The newly formed building blocks are then ready to be incorporated into their respective filaments, F-actin and microtubules. CCT may localize to pre-existing actin filaments, where it can help in the folding of mRNAs that would then be bound to these filaments, creating local gradients of protein concentration. Organelles and cell components are not depicted to scale. Inset, CCT organization and ATP consumption.

The actin-tubulin interconnection seems to be prior to the formation of their respective filaments, initiating at their folding upon *de novo* synthesis. Present in all eukaryotes, the cytosolic group II chaperonin CCT is an oligomer of about 1 MDa composed of eight different subunits (CCT1-8) that organize into a barrel-like structure formed by two back-to-back rings (Skjærven et al., 2015), with an already defined arrangement (CCT1-4-2-5-7-8-6-3, with CCT2 and CCT6 establishing homotypic, inter-ring interactions; Figure 1; Leitner et al., 2012; Kalisman et al., 2013; Chagoyen et al., 2014). However, during the oligomerization process, CCT microcomplexes can be observed (Sergeeva et al., 2019). The rings operate sequentially to assist in the folding of different clients (e.g., tubulin and actin monomers) upon their synthesis at the ribosome (Willison, 2018). CCT accumulates at the centrosomes of activated T cells (Martin-Cofreces et al., 2020), together with other complex oligomers such as the proteasome (Martin-Cofreces et al., 2020), also found in B cells (Ibañez-Vega et al., 2019). The proteasome is involved in degradation of ubiquitinated and unfolded proteins at the centrosomes of different cell types, which has been linked to the control of centrosome function (Freed et al., 1999; Vora and Phillips, 2016).

## Chaperone Activities of CCT: Folding and Others

Actin and tubulin are major clients of CCT, which is considered to have co-evolved with these two components of the cytoskeleton, facilitating their current structural and molecular mechanisms. Native actin and tubulin are absolutely essential for cells, which makes the CCT oligomer an indispensable complex for cell viability (Liu et al., 2005). Many studies performed in yeast involving genetic deletion of individual subunits have shown growth defects and a loss of viability. Cells presented aberrant morphology and abnormally large sizes (Willison, 2018). The different CCT subunits can exert independent functions in cells (Vallin and Grantham, 2019); thus, the effects observed after simultaneous knockdown of several subunits probably correspond to the holoenzyme activity, whereas differences detected upon silencing of single subunits may be attributed to that particular component.

Studies on the CCT interactome are allowing the discovery of not only potential clients for CCT (either for complete or intermediate folding), but also regulatory proteins or proteins that are controlled by CCT (Dekker et al., 2008; Yam et al., 2008). The functions of obligate clients are linked to CCT folding activity: if the chaperonin fails to properly assist the folding of these substrates, loss of function effects could occur. Consequently, an excess of substrate may provoke an excess of unfolded forms of other substrates, (i.e., competition with other substrates), leading to toxicity through protein aggregation. Actin and tubulin connect CCT to any process that depends on the function of microtubules and actin filaments (Sternlicht et al., 1993). Since actin and tubulin are major clients for CCT, probably due to their abundance and affinity (Willison, 2018), their expression likely regulates the quantity of CCT available for other substrates, thus linking this process to normal cell metabolism and cell cycle progression. Other CCT client proteins include members of the WD40 family, which contain tandem copies of a 40-amino acid repeat (WD40 motif), several of which form a β-propeller structural domain. The cell cycle regulators Cdh1 and Cdc20 belong to this family of proteins and are also folded by CCT (Camasses et al., 2003). The two proteins play critical roles as adaptors for the anaphase promoting complex/cyclosome (APC/C), a ubiquitin ligase regulating cell cycle progression (Camasses et al., 2003). CCT may also impact cell biology through the folding of another WD40 protein, mLST8 (a subunit of mTOR complexes 1 and 2; Cuellar et al., 2019), which in turn regulates protein synthesis through the ribosomes and therefore, the flux of CCT clients. mTOR is activated by TCR stimulation and CD28 co-stimulation, increasing the eukaryotic translation initiation factor 4E (eIF4E) binding proteins (4E-BP1, 2, and 3) and the p70 ribosomal S6 kinase (S6K) activity. S6K phosphorylates the S6 ribosomal subunit to initiate protein synthesis (Myers et al., 2019). S6K also phosphorylates CCT2 subunit, providing a link between mRNA translation and folding (Abe et al., 2009). Another WD40 protein is Gβ, a component of the Gβ-Gγ signaling heterodimer complex. Here, CCT not only plays a role in the folding of Gβ, but also in the stabilization of the Gβ-Gγ complex (Plimpton et al., 2015).

## Regulation of the Actin Cytoskeleton by CCT

Despite our understanding of how a multitude of extracellular stimuli trigger pathways that lead to actin polymerization/depolymerization, and the detailed molecular mechanism of local actin nucleation (Pollard, 2019), some challenges remain to be addressed. There is a need to learn about the mechanisms that control newly assembled F-actin into higher order structures such as stress fibers, filopodia, and other bundles including short filaments near membrane-resident receptors. Indeed, additional work on the regulatory and functional mechanisms of F-actin disassembly is required to explain its rapid pace in cells.

A pending question about actin dynamics regards the maintenance of monomer availability in cells. Newly synthesized actin requires CCT to adopt its native structure; a transient 90% silencing of CCT by siRNA only slightly affected actin synthesis, but reduced the amount of native actin and therefore cellular motility (Grantham et al., 2006). There is limited evidence of the regulation of actin filament homeostasis by synthesis and degradation of monomers, although such a process (involving specific degradation and isoform replacement of conventional actin through the proteasome) has been described in *Chlamydomonas* during stress adaptation (Onishi et al., 2018). The degradation of γ-actin by N-terminal arginylation upon removal of the N-terminal methionine depends on its ubiquitination and proteasome processing, and relies on slow translation and exposure of a Lys residue. Arginylated β-actin is more stable than the unmodified protein (Zhang et al., 2010). These modifications may heavily impact the ability of cells to expand their lamella at the front edge and migrate (Kashina, 2006). The majority of actin in lymphocytes corresponds to the β isoform (about 80%), whereas the remaining 20% is γ-actin, providing a mechanism to regulate actin availability through the proteasome. The role of the proteasome has been tested in T and B cells using different inhibitors during the establishment of synaptic contacts. The use of both MG132 and epoxomycin in activated B cells has shown effects on actin remodeling around the centrosome, which would prevent centrosome detachment from the nucleus and translocation to the synapse (Ibañez-Vega et al., 2019). All these inhibitors also showed effects on the tubulin cytoskeleton (Didier et al., 2008; Poruchynsky et al., 2008; Meregalli et al., 2014). In T cells, the highly selective proteasome inhibitor bortezomib increased tubulin dynamics at the centrosome area near the IS (Martin-Cofreces et al., 2020), while its effect on the actin cytoskeleton is not yet reported. Additional studies are needed in order to understand whether these outcomes are a direct consequence of the lack of proteasome activity on specific substrates, or rather the result of a broad inhibition of other cell components with similar selectivity, such as calpains and cathepsin B. On the other hand, β-actin synthesis increased in centrosomes upon TCR activation, but a 40% reduction of CCT levels did not impact the total β-actin levels in T cells (as observed in other cell systems with increased reduction of the oligomer; Grantham et al., 2006). CCT reduction neither prevents the extension of the actin lamella nor adhesion to the APC, and phosphorylation of the myosin light chain is conserved (Martin-Cofreces et al., 2020).

The CCT complex co-sediments with F-actin in *in vitro* assays, where the initial rate of actin polymerization at the plus-ends is reduced, although F-actin formation is not prevented (Grantham et al., 2002). Indeed, although CCT does not assist gelsolin folding, it binds to its Ca^2+^-activated form (Svanstrom and Grantham, 2016), which may control F-actin elongation through its severing and capping activity. The *in vivo* consequences of these interactions are under study. With regard to individual subunits, a reduced level of the CCT5 subunit narrows the cell shape and reduces the area of adhesion to substrate (Brackley and Grantham, 2010). Isolated CCT subunits are found near F-actin bundles (Brackley and Grantham, 2010), and over-expression of CCT4 induces cellular protrusions and filopodia (Spiess et al., 2015), which may reflect the individual roles of these proteins in the cell. Whether these individual CCT subunits or the oligomer play such a role in the IS deserves future experimentation.

An interesting feature of actin mRNA translation is that the zip code region at the 3′UTR of β-actin mRNA regulates its localization at the leading edge in migrating cells, in a serum-dependent manner (Kislauskis et al., 1997). Research performed on the contribution of newly synthesized β-actin to actin dynamics has suggested that it is unlikely that the calculated 6.5% of this *de novo* actin will significantly contribute to the rate of global actin polymerization at the cell front edge (Shestakova et al., 2001). However, this process performed in a restricted volume would increase the limiting monomer concentration, establishing a major location for actin polymerization. The delocalization of β-actin mRNA alters the sites and rate of cell protrusion (Shestakova et al., 2001). In this regard, the requirement of F-actin to localize its own mRNA to the front edge may also be considered a local mechanism of control by *de novo* synthesis (Sundell and Singer, 1991) in which CCT would take part. The cytoskeletal organization at the IS can be compared to that of the front-edge during migration (Figure 1). The interaction of tubulin and vimentin mRNAs with F-actin has also been described (Singer et al., 1989). Conceivably, this mechanism regulating the half-life of these messengers and the timing of their translation would constitute another regulatory step between the cytoskeletons.

The above model would be further supported if *de novo*-synthesized G-actin had a different effect on the polymerization rate of F-actin compared to the G-actin already present, either by its own molecular structure or by its affinity for nucleation complexes. G-actin may be subjected to post-translational modifications (PTMs) such as S-nitrosylation of β-actin on Cys374, which may change actin’s molecular structure and properties (Garcia-Ortiz et al., 2017). This oxidation is relevant to regulate the ability of actin to polymerize and depolymerize at the IS, based on its interaction with profilin (Garcia-Ortiz et al., 2017). Although actin modifications have not been extensively studied in the context of synapses, more than 140 PTMs have been described in eukaryotic actin sequences. Some of them are quantitative and reversible, whereas others are infrequent and affect a minority of the actin pool. Specifically, N-terminal acetylation, arginylation and novel oxidation, phosphorylation, ubiquitination, and SUMOylation sites have been identified in recent years, some of them by global proteomics analyses (Varland et al., 2019). All these modifications may impact the native structure and stability of the protein, and constitute a growing field of research to be developed in the future.

## Tubulin Regulation by CCT

Tubulin molecules are the building blocks of the structure that controls cell shape and dynamics, and can originate from a large number of genes and isotypes. These diverse gene products organize into heterodimers such as αβ-tubulin (which forms microtubules) and γ-tubulin, which is found in specific structures such as the centrioles within centrosomes and in γ-TURC complexes, used as seeds to initiate microtubule polymerization at the pericentrosomal matrix and Golgi apparatus (Bettencourt-Dias and Glover, 2007; Martin-Cofreces and Sanchez-Madrid, 2018). There are other tubulins including ε-, ζ- and δ-tubulin, which are less studied and present only in some eukaryotes. Mutations in tubulin genes cause multiple human cortical malformations (tubulinopathies) that include microcephaly, lissencephaly, dysmorphic basal ganglia and polymicrogyria (Bahi-Buisson et al., 2014). Synthesis and folding of the heterodimers is a precise and complex process that requires the action of the prefoldin complex, a cochaperone that binds to the nascent polypeptide and transfers it to CCT to be folded (Llorca et al., 2000). The folded protein later depends on cooperation of different tubulin binding co-factors (TBCs) that specifically bind to α- or β-tubulin, helping in the formation of αβ-heterodimers and their incorporation into microtubules (Lopez-Fanarraga et al., 2001).

Tubulin synthesis is regulated by the cell cycle, increasing during the S phase to facilitate organization of the mitotic spindle. It is also self-regulated by its own quantity in the cells, thereby maintaining a constant pool of available heterodimers for microtubule dynamics (Baker, 1993; Brackley and Grantham, 2009; Willison, 2018). The effects of different inhibitors of protein synthesis and the proteasome have been studied in diverse cell systems. During IS formation, active tubulin synthesis is driven by TCR activation, providing increased availability of soluble heterodimers for polymerization at the centrosome. This local increase in tubulin concentration would burst microtubule polymerization from the centrosome, facilitating the radial array formed at the immune synapse (Figure 1). The inhibition of protein synthesis in T cells through the chemical inhibitor cycloheximide prevented tubulin dynamics during T cell activation, as did a 40% reduction in CCT levels by siRNA (Martin-Cofreces et al., 2020). At this level of expression, total cellular quantities of actin and tubulin were unaffected, whereas a 90% reduction in CCT showed diminished levels of tubulin (Grantham et al., 2006). Despite the great increase in soluble heterodimers, polymerization from the centrosome was strongly decreased, both in terms of the number of new microtubules and the rate of incorporation. In contrast, treatment with the highly selective proteasome inhibitor bortezomib increased the rate of polymerization from the translocated centrosome, reducing soluble cytosolic heterodimers. The use of other proteasome inhibitors, such as MG132 and epoxomycin during IS formation has focused on the study of F-actin at the centrosome area, showing an inhibition of the centrosome’s translocation to the IS in B cells (Ibañez-Vega et al., 2019). MG115, PS-341 and epoxomycin treatments in HeLa cells increased the amount of proteins such as γ-tubulin, dynactin, ninein and PCM-1 at the centrosomes, generating an enlargement of this organelle. Epoxomycin prevented the radial array of microtubules in interphase U2OS cells after nocodazole treatment without preventing centriole conformation, as observed through electron microscopy (Didier et al., 2008). In contrast, bortezomib treatment increases microtubule dynamics (Poruchynsky et al., 2008; Meregalli et al., 2014). These apparently contradictory results warrant further research to understand the role of the proteasome on cytoskeleton dynamics at the centrosome.

An intriguing fact is that the centrosome of T cells with reduced levels of CCT was not as distant from the IS as should be expected, as observed by soft-X-ray cryocorrelative microscopy, with a spatial resolution of about 50 nm (Martin-Cofreces et al., 2020). A recent report describes that kinesin-4 KIF21B limited the growth of microtubules shortly after TCR activation, allowing translocation of the centrosome (Hooikaas et al., 2020), which may correlate to no specific defects in centrosome translocation to the IS in T cells with diminished microtubule growth due to cycloheximide treatment or reduced CCT levels. Instead, it might be the result of different doses and timing of the different treatments, leading to diverse responses or a lack of effect. In this regard, the internal ultrastructure of the centrosome at the IS is affected by a reduction in CCT levels. The centrioles show a different orientation inside the centrosome with respect to the IS plane once the TCR is activated, which is prevented by CCT knockdown (Martin-Cofreces et al., 2020). The orientation of the centrioles seems to allow the oriented polymerization of microtubules toward the IS, even if the centrosome is not close to it. This piece of evidence may imply further biological consequences than the actual position of the centrosome in the cell, and warrants future research on how this orientation is regulated, and whether the centrioles can act as a sensor for “up and down” positioning in cells.

**FIGURE 2:**
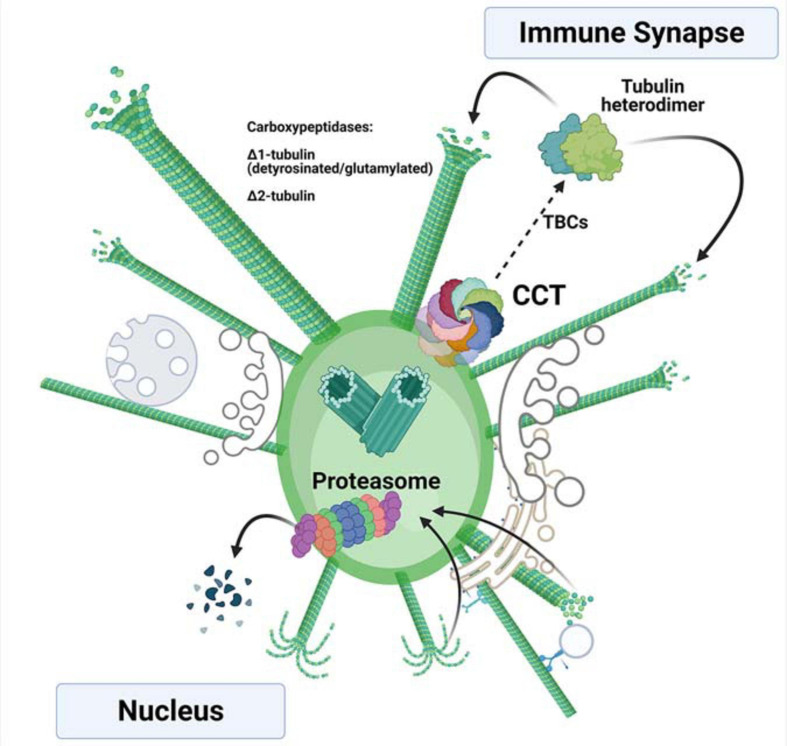
Tubulin synthesis and degradation at the centrosome of activated T cells. Newly synthesized α- and β-tubulin are assisted in their folding by the chaperone CCT, forming heterodimers that can be incorporated into nascent microtubules with the assistance of different tubulin-binding co-factors (TBCs). The incorporated heterodimers can then be post-translationally modified by carboxypeptidases that delete the C-terminal tyrosine (Δ1-tubulin) and the glutamic acid (Δ2-tubulin). These modifications take place in the microtubules. The depolymerized, post-translationally modified heterodimers can be then proteolyzed by the proteasome upon depolymerization. The rapid synthesis and degradation of tubulins enable availability of fresh heterodimers for polymerization. If the CCT chaperonin and proteasome localize asymmetrically inside the centrosome, they can act in different orientations (i.e., the nuclear and immune synapse sides), allowing directionality of the polymerization.

Additionally, a specific equilibrium between tubulin synthesis and degradation may also be required to allow correct microtubule dynamics, which might be dependent on the incorporation of building blocks into the polymer and the PTMs observed in tubulin (Figure 2). The de-tyrosination of tubulin at its C-terminus (Δ1-tubulin) has been observed during IS formation, with increased localization at the centrosome (Andres-Delgado et al., 2010). This PTM is also observed in soluble heterodimers upon TCR activation, concomitant with increases in Δ2-tubulin (a variant that lacks the C-terminal tyrosine and glutamic residues; Martin-Cofreces et al., 2020), which has been proposed to be a form of tubulin targeted for degradation (Paturle-Lafanechere et al., 1994). Reduced protein synthesis either by cycloheximide treatment or CCT knockdown prevents not only the incorporation of heterodimers into microtubules, but also the aforementioned PTMs, accompanied by increasing microtubule acetylation (Martin-Cofreces et al., 2020). Deacetylation of microtubules driven by HDAC6 is observed shortly after TCR activation, indicating a transient increase in dynamic microtubules during T cell reorganization (Serrador et al., 2004). Over-expression of HDAC6 prevents centrosome translocation (Serrador et al., 2004), and its knockdown decreases the distance of the centrosome to the IS (Nunez-Andrade et al., 2016), which would correspond to a sustained increased acetylation, as observed in T cells with reduced CCT levels (Martin-Cofreces et al., 2020). Indeed, knock-down of Aurora A, a Serine/threonine kinase that promotes microtubule growing from the polarized centrosome in synaptic T cells, does not affect centrosome polarization, but alters microtubule growing at the IS (Blas-Rus et al., 2017). Aurora A promotes microtubule polymerization from the centrosome (Terada et al., 2003). CCT depletion or CEP55 knock-down decrease Aurora A at the protein level and prevents ciliary disassembly; in absence of Aurora A, cilia are longer with increased acetylated microtubules (Zhang et al., 2021). Also, Aurora A phosphorylates and activates HDAC6 to allow ciliary dissasembly (Pugacheva et al., 2007). A similar mechanism can be acting at the IS as the cilia and the IS share components and features (Cassioli and Baldari, 2019). Deacetylation of microtubules may be required to disassemble the initial microtubular network and to allow the microtubule-organizing activity of the centrosome. Those results support that defects in microtubule growth show a lesser effect on centrosome translocation than an excess of polymerization during IS formation (Hooikaas et al., 2020). The lack of microtubule polymerization in T cells with reduced CCT levels might prevent tubulin clearance by the proteasome, which would be mainly loaded with Δ2-tubulin (Figure 2). The decrease in Δ1-tubulin allows rapid depolymerization at plus ends of microtubules by action of kinesin 13, which binds preferentially to tyrosinated tubulin (Peris et al., 2009). This would be observed as a reduced polymerization rate in TIRFm assays in terms of the number of microtubules and polymerization speed at the IS. Together, these events may result in an accumulation of unmodified tubulin heterodimers, as indeed has been observed (Martin-Cofreces et al., 2020). The lack of tubulin dynamics induces defects in the structure of the IS, including mitochondrial disorganization and failures in cell respiration. These effects may also be supported by decreased mTOR activity (Cuellar et al., 2019).

## Is There Any CCT Connection Driving the Synchronization of Cytoskeleton Dynamics?

It is remarkable to notice that actin dynamics, which are more readily observed upon TCR activation (from ms to s) than tubulin dynamics (from s to min), are less dependent on the *de novo* synthesis of its constituent G-actin. This effect has been observed in different cell types (Grantham et al., 2006; Willison, 2018; Martin-Cofreces et al., 2020) and is dependent on the amount of functional CCT available. During *in vitro* translation assays with rabbit reticulocyte lysates, CCT was required for folding of actin into its native form (Garcia-Ortiz et al., 2017; Willison, 2018). However, once folded, actin seems to be more stable than tubulin, which requires a much more complex folding process and cooperation between multiple co-factors to polymerize and depolymerize (Lopez-Fanarraga et al., 2001; Kortazar et al., 2007). Even if small changes in CCT do not result in changes in the amount of actin or tubulin in resting cells, CCT’s boost of tubulin polymerization may be extremely important for the cytoskeletal reorganization needed during the dramatic structural changes that occur in processes such as mitosis, leading edge extension in migrating cells, or IS formation. The differing requirements for tubulin and actin synthesis to increase the critical or limiting concentration may determine in these scenarios the timing of local polymerization for each filament type.

## Concluding Remarks

An attractive hypothesis is that during T cell activation the centrosome may arrange the CCT chaperonin and the proteasome *asymmetrically*, in coordination with the change in reciprocal orientation of the centrioles, thereby allowing major depolymerization and degradation of tubulin on the “nucleus side” and synthesis and polymerization on the “IS side” (Figure 2). Such a hypothesis would explain how the proteasome is unequally distributed/divided between the mother and daughter cells during asymmetric division of CD8 + T cells to generate memory T cells; the correct localization of the centrosome seems to be required for this process (Chang et al., 2011; Martin-Cofreces et al., 2014). The use of inhibitors in these experiments is challenging, since their effect is global and they would be expected to act firstly at sites where the actin or tubulin cytoskeletons respond rapidly to TCR activation, such as the microvilli or the lamella at contact sites with the APC (Figure 1), acting only secondarily at the centrosome. Imaging methods allowing single-particle localization combined with CRISP/Cas technology to substitute CCT subunits in cells may help to study these events. The increase in spatial resolution provided by emergent microscopy technologies, such as cryocorrelative microscopy and subsequent cryoelectron tomography and subtomogram averaging will allow localizing this kind of complexes, thus helping to better understand the biological processes described above.

## Author Contributions

NM-C: conceptualization, funding acquisition, image composition (Figures 1, 2), and writing (original draft, review and editing). FS-M and JMV: conceptualization, resources, funding acquisition, and writing (original draft, review, and editing). All authors contributed to the article and approved the submitted version.

## Conflict of Interest

The authors declare that the research was conducted in the absence of any commercial or financial relationships that could be construed as a potential conflict of interest.
